# Satisfactory medium-long term patient reported outcomes after laparoscopic single-mesh sacrohysteropexy

**DOI:** 10.52054/FVVO.14.2.017

**Published:** 2022-07-01

**Authors:** F Dökmeci, Y.E. Şükür, Ş.E. Çetinkaya, M.M. Seval, B Varlı

**Affiliations:** Ankara University School of Medicine, Department of Obstetrics and Gynecology, Ankara, Turkey

**Keywords:** Laparoscopy, laparoscopic hysteropexy, patient reported outcome, uterine prolapse

## Abstract

**Background:**

There is scarce information on the effectiveness of the laparoscopic single mesh sacrohysteropexy (smSHP). Attachment of a single sheet of flat mesh posteriorly to the cervix provides less mesh use and a less invasive distal mesh fixation.

**Objectives:**

To assess medium to long-term follow-up results of patients who underwent laparoscopic smSHP utilising a less invasive technique with single sheet flat mesh.

**Materials and Methods:**

In the present retrospective cohort study, the data of 71 women who underwent laparoscopic smSHP for apical uterine prolapse with or without colporrhaphy (anterior and/or posterior) at the urogynaecology unit of a university hospital between January 2008 and January 2020 was reviewed. Data was collected on demographics, presenting symptoms, preoperative findings, surgery, and postoperative outcomes.

**Main outcome measures:**

Medium to long-term patient-reported outcomes.

**Results:**

The median age of the study population was 44 years. Median follow-up duration was 5 years (1-12). Symptomatic recurrence over time and repeat surgery rates were 13.1% and 3.1% respectively. Comparison of the pre-operative and medium to long-term evaluation scores of the pelvic floor distress inventory-20 (PFDI-20) and assessment of the patient global impression of improvement (PGI-I) revealed long-standing improvement in pelvic floor dysfunction.

**Conclusions:**

Laparoscopic smSHP appears to be successful and safe with low recurrence and complication rates and provides satisfactory patient reported outcomes.

**What's new?:**

Medium to long-term patient-reported outcomes based on PFDI-20 and PGI-I surveys are satisfactory following smSHP.

## Learning objective

Uterine preservation surgeries have gained popularity due to potential benefits including avoidance of hysterectomy related risks, maintenance of childbearing potential, improved lower urinary tract symptoms (LUTS), preservation of sexual function, protective effect on ovarian function, supportive effects on the other compartments, and patient satisfaction (Betschart et al., 2017; Kow et al., 2013; Aslam et al., 2017). Laparoscopic procedures have the significant advantages of shorter hospital stay, less blood loss and reduced rates of bowel complications (Mueller et al., 2016; Nosti et al., 2014). In the most recent meta-analysis, Meriwether et al. (2014) found that laparoscopic sacrohysteropexy (LSHP) had lower recurrent prolapse symptoms than open SHP. The recent National Institute for Health and Care Excellence (NICE) guideline (NICE, 2019), which is specific to UK, recommended the inclusion of the option of SHP with mesh for women with uterine prolapse who wish to preserve their uterus or have no preference about preserving their uterus.

A wide variety of potential complications exist with mesh-augmented pelvic organ prolapse (POP) surgeries including mesh exposure, pain, infection, bleeding, dyspareunia, and mesh contracture (Ellington and Richter 2013; Izett-Kay et al., 2020). Regarding mesh-associated complications, the International Federation of Gynaecology and Obstetrics (FIGO) working group recommends limiting the amount of mesh (Betschart et al., 2017). Indeed, it is worthy to mention that there has been a global scrutiny of all mesh-augmented surgery within the last decade (Ugianskiene et al., 2019).

In the literature, information has accumulated on dual-mesh systems where the distal end of the mesh is placed on both posterior and anterior aspects of the cervix and vaginal wall (Jefferis et al., 2017; Lone et al., 2018; Kupelian et al., 2016). Recently, Jan et al. (2018) described their simplified laparoscopic SHP technique without dissection of the broad ligament and bladder and reported three mild recurrences in the short term. Considering the recommendations to utilise mesh as little as possible, this technique seems to be a reasonable option. However, data on the long-term follow-up results of patients who underwent laparoscopic single mesh sacrohysteropexy (smSHP) is still limited. The aim of this study was to assess medium to long-term patient reported outcomes and recurrence rates of laparoscopic smSHP procedure implemented since 2008 with a standardised technique in a single centre.

## Materials and Methods

### Patient Selection

In the present retrospective cohort study, the data of women who underwent laparoscopic smSHP for uterine prolapse at the urogynaecology unit of Ankara University School of Medicine, Department of Obstetrics and Gynaecology between January 2008 and January 2020 was reviewed. The study was approved by the Ethical Committee of Ankara University School of Medicine (Approval # 07-366-17, date: 10.04.2017). All the data was assessed for eligibility from the urogynaecology unit database, recorded both electronically and on paper files. Two researchers (YEŞ and BV) undertook the chart reviews blinded to each other to ensure accuracy and to avoid recording bias. Women with stage 3 or 4 uterine prolapse, i.e., those with a C point >+1 (POP-Q stage 3 or 4), who fulfilled the Turkish validated Pelvic Floor Distress Inventory-20 (PFDI-20) (Balkanlı-Kaplan et al., 2012) and Patient Global Impression of Improvement (PGI-I) at the last follow-up visit of at least 12 months were included. In case of non-attendance, women were phone-called, and the questionnaires were either posted or sent via e-mail.

Exclusion criteria were absence of adequate pre-/postoperative patient evaluation records and loss to follow-up in the past five years.

### Preoperative assessment

All patients were examined by at least one senior consultant at the urogynaecology unit. Pelvic Organ Prolapse Quantification (POP-Q) staging was performed in the lithotomy position, as described by the International Continence Society Committee on Standardization of Terminology (Haylen et al., 2010).

Symptoms of prolapse were identified using the Turkish validated PFDI-20 questionnaire. During the preoperative shared decision–making process, all patients were informed about all surgical approaches and techniques with pros and cons for uterine prolapse repair with or without uterine preservation options. An informed consent regarding the intra- and post-operative risks and complications specific to the surgical technique and mesh utilisation was taken from all patients. In addition, all patients who had lower urinary tract symptoms (LUTS) were evaluated with single voiding cycle ambulatory urodynamics, which has been used as the primary urodynamic investigations in our clinical setting since 2011.

### Standardised Surgical Technique

All operations were performed by one of three senior consultants at the urogynaecology unit (FD, ŞEÇ, MMS). The operation is performed under general anaesthesia in the lithotomy position with all patients wearing gradual compression stockings. A uterine manipulator is inserted after induction of general anaesthesia. After insufflation with the Veress needle, a 10-mm 0° telescope is inserted through the umbilicus and accessory trocars are placed under direct visualisation to bilateral iliac fossa and left upper quadrant.

**Figure 1:**
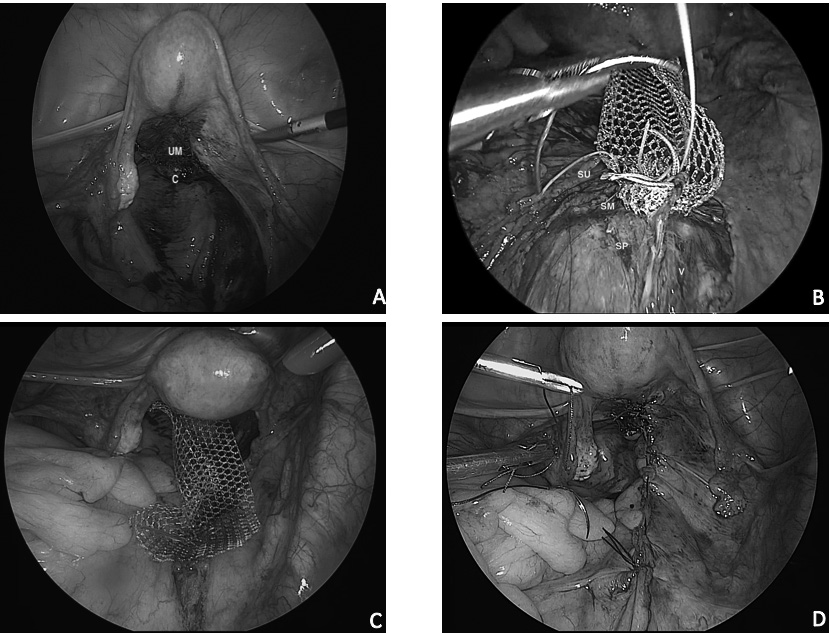
Description of the surgical technique. (A) Mesh attachment site at the uterus. (B) Mesh attachment site at the sacrum. (C) Mesh fixated to the uterus and the sacrum. (D) Final view of the surgical site after closure of peritoneum. C: Cervix, SM: Mesh attachment site at the sacrum, SP: Sacral promontory, SU: Non-absorbable sutures, UM: Mesh attachment site at the uterus, V: Presacral veins.

Exposure to the sacral promontory is eased by positioning the patient on Trendelenburg’s position and by retracting the bowels either via sutures or via the T-lift surgical retractor (VECTEC, Hauterive, France). The peritoneum is opened over the sacral promontory and the anterior longitudinal ligament is exposed. Then, the incision is directed at the right pelvic sidewall between the ureter and rectum, toward the pouch of Douglas and the serosal surface of the cervix, up to the cervico-uterine junction using scissors (Figure 1A).

A type-1 monofilament macroporous polypropylene mesh (Gynecare Gynemesh PS Nonabsorbable Prolene Soft Mesh, Ethicon, Johnson&Johnson, USA or Parietene; Covidien, Trevoux, France) is then used for uterine suspension. A single mesh measuring 15x3 cm is first fixed to the anterior longitudinal ligament at the level of S1 or 2 with two to four, number 0 polypropylene (Prolene, Ethicon, Johnson&Johnson, USA) or polyethylene terephthalate sutures (Ethibond, Ethicon, Johnson&Johnson, USA) (Figure 1B). Care is taken not to damage the presacral and iliac vessels and nerve fibres of the superior hypogastric plexus. Uterine anchoring of the mesh is done on the posterior aspect of the cervix at the level of cervico-uterine junction while passing through the uterosacral ligaments (USL) with three to four number 0 polyglactin 910 sutures (Vicryl, Ethicon, Johnson & Johnson, USA) (Figure 1C). The broad ligament and the anterior aspect of cervix are not dissected. The C-point is re-positioned to be at < -1 and considering the potential of increased tension due to mesh shrinkage, over-correction to stage 0 is avoided. The peritoneum is closed with a 2/0 polyglactin 910 suture (Vicryl, Ethicon, Johnson & Johnson, USA) or a locking 2/0 barbed suture (V-Loc, Covidien, Mansfield, USA) to cover the mesh (Figure 1D). Vaginal wall repair is performed concomitantly in cases of stage 3 or 4 anterior and/ or posterior prolapse (Ba≥+2; Bp≥+2). Concomitant vaginal prolapse surgeries and/or anti-incontinence procedures are performed before the laparoscopic approach in order to facilitate the vaginal approach.

### Follow-up

The first post-operative follow-up visits are done at 1 and 3 months after surgery. At annual follow-up visits, all attending patients are examined by a senior consultant at the urogynaecology unit. Pelvic floor symptoms were evaluated with the PFDI- 20 questionnaire, and the PGI-I scale was used to assess patient satisfaction; all patients were asked to complete both questionnaires at each follow-up assessment. Symptomatic prolapse recurrence was 15 identified using question 3 of the validated Turkish version of PFDI-20 questionnaire: ‘Usually have a bulge or something falling out that you can see or feel in your vaginal area?’. In cases of non- attendance, women were phone-called, and the questionnaires were either posted or sent via e-mail. Anatomical recurrence could be evaluated in only attending patients using the POP-Q system and was defined as C ≥+1 or Ba ≥+1.

### Statistical Analysis

Data analyses was performed using SPSS Version 21.0 (IBM Corporation, Armonk, NYC, USA). The normality of distribution of variables was evaluated by Shapiro-Wilk test. According to the results, non-parametric tests were preferred. Descriptive statistics of continuous variables were compared between groups using Mann-Whitney U test. The Chi-square test or Fisher’s exact test (when chi-square test assumptions do not hold due to low expected cell counts) were used to compare categorical variables between groups. Continuous variables were presented as median and minimum- maximum values, whereas categorical variables were presented as number and percentage. A P value of <0.05 was considered statistically significant.

## Results

### Patient characteristics and baseline data

**Figure 2:**
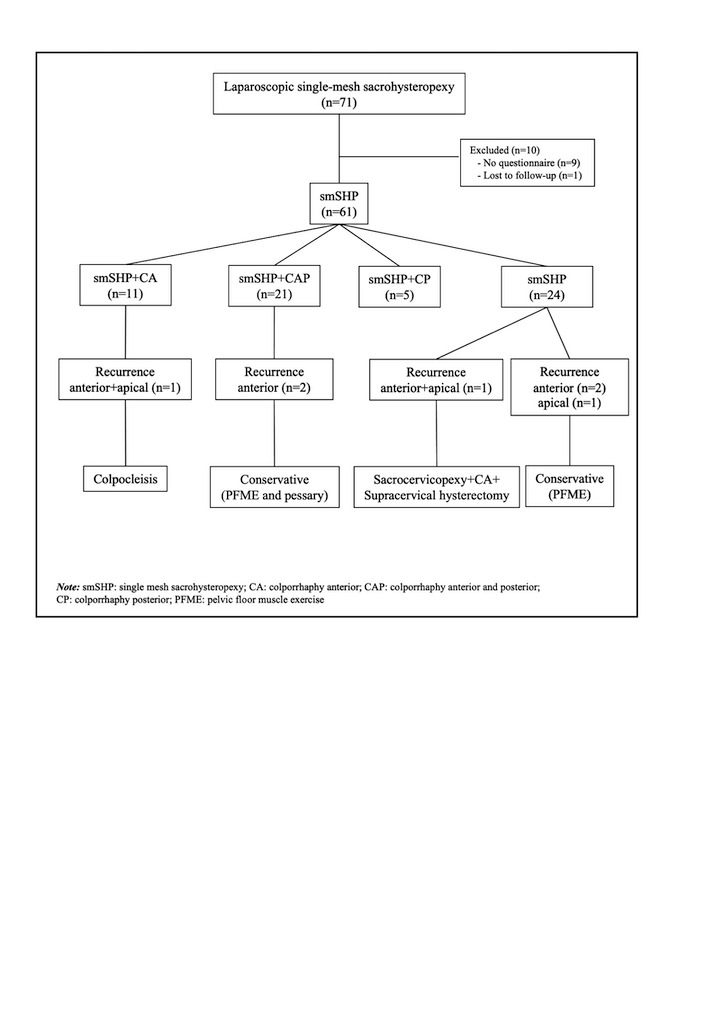
Flow-chart of the laparoscopic single mesh sacrohysteropexy cases with or without concomitant anterior and/or posterior repair.

Out of 71 laparoscopic smSHP patients, 10 were excluded due to absence of adequate pre-/post-operative questionnaires (n=9), and loss to follow-up in the past five years (n=1). As a result, 61 patients were found to be eligible for final analysis (Figure 2).

The median age was 44 years (29-82 years). Among all, 37.7% were postmenopausal and the median number of parities was 2 (1-5) (Table I). Of the 61 patients, six (9.8%) reported previous prolapse surgery, 16 (26.2%) had constipation and 21 (34.4%) had LUTS. The findings obtained from ambulatory urodynamic studies of patients with LUTS were summarised in Table II. None of the patients had future childbearing planned at the time of surgery.

**Table I:** Preoperative demographic characteristics of the study population (N=61).

Age, years	44 (29-82)
Body mass index, kg/m^2^	28 (24-44)
Parity, n	2 (1-5)
Maximal birth weight, grams	3500 (2500-4500)
Previous abdominal surgery, n (%)	13 (21.3)
Previous POP repair, n (%)	6 (9.8)
Chronic disease, n (%)	18 (29.5)
Smoker, n (%)	10 (16.4)
Post-menopausal, n (%)	23 (37.7)

Note: POP-Q, pelvic organ prolapse quantification. Continuous data are presented as median (min-max) values, categorical data are presented as frequency (percentage).

**Table II:** Findings of ambulatory urodynamics in patients who had complicated lower urinary tract symptoms (N=21).

Duration of the test, minutes	77 (45-273)
Voided volume, mL	253 (90-600)
Bladder capacity, mL	370 (160-670)
Maximal flow rate, mL/sec	22 (4-54)
P_det_Q_max_, cmH_2_O	34 (18-223)
Post-void residual volume, mL	60 (10-275)
Pad test, gram	0 (0-108)
Detrusor overactivity, n (%)	12 (57.1)
Pure stress incontinence, n (%)	2 (9.5)
Mixed urinary incontinence, n (%)	3 (14.3)
Urgency, n (%)	5 (23.8)

Note: Continuous data are presented as median (min-max) values and categorical data are presented as frequency (percentage).

### Perioperative data

The median operating room time, defined as the period from patient arrival to operating room to the transfer to recovery following extubation was 120 minutes (90-330). Forty-five patients (73.8%) underwent concomitant surgical procedures (Table III).

**Table III:** Intra-operative and post-operative characteristics of the study population.

Intra-operative	
Operating room time, min	120 (90-330)
Concomitant surgeries, n (%)	
- Colporrhaphy anterior	32 (52.5)
- Colporrhaphy posterior	26 (42.6)
- Mid-urethral sling	2 (3.3)
- Supracervical hysterectomy	4 (6.6)
- Tubal ligation	6 (9.8)
- Myomectomy	1 (1.6)
- Salpingo-oophorectomy	1 (1.6)
- LEEP+Cystoscopy	1 (1.6)
Conversion to laparotomy	1 (1.6)
Post-operative period	
Early post-operative complication (<30 days), n (%)	4 (6.6)
Follow-up duration, years	5 (1-12)
Symptomatic POP recurrence, n (%)	8 (13.1)
Isolated anterior compartment recurrence	4 (6.6)
Repeat surgery for apical prolapse, n (%)	2 (3.1)
Time to symptomatic prolapse recurrence, years	1 (1-3)
Time to repeat surgery for apical prolapse, years	2 (1-3)

Note: LEEP: loop electrosurgical excision procedure; POP:pelvic organ prolapse. Continuous data are presented as median (min-max) values and categorical data are presented as frequency (percentage).

Among all, 37 (60.7%) underwent anterior and/ or posterior vaginal wall repair. There were no intraoperative complications except the need for conversion to laparotomy in one patient (1.6%) who suffered from intraoperative respiratory problems.

Four patients (6.6%) experienced early onset (within 30 days after operation) complications: trocar site infection, abdominal wall hematoma from accessory trocar insertion site, pulmonary thromboembolism and pyelonephritis which progressed to urosepsis. Medical treatment or conservative follow-up were successful for all four cases.

### Follow-up data

The median follow-up duration was five years (1-12 years). All patients fulfilled PFDI-20 and PGI-I questionnaires at the time of this audit. Of the 61 patients, eight (13.1%) reported symptomatic recurrence, identified according to question 3 of the PFDI-20. Among those, seven were evaluated and confirmed to have anatomical recurrence (Figure 2). One patient did not attend the physical examination and anatomical recurrence could not be confirmed for this patient. Four patients had stage 1 and 2 (Ba≤+1) anterior prolapse and one patient had stage 2 (C≤+1) apical prolapse and were all treated conservatively with pelvic floor muscle exercises. Two of them who had used pessaries prior to surgery, preferred to use pessaries after postoperative anterior compartment recurrence, and both were satisfied. Two patients had stage 3 or 4 apical and anterior and/or posterior prolapse recurrences and underwent repeat surgeries (3.2%); one underwent laparoscopic sacro-cervicopexy with anterior repair and concomitant supra-cervical hysterectomy and the other preferred colpocleisis.

The median duration between the initial and repeat surgeries was 2 years (1-3 years). Among all recurrence patient (n=7; 13.1%), four had isolated anterior compartment prolapse (6.6%). There was no major mesh related short- or long- term complications including pain, extrusion, re- operation or adhesions.

According to the PGI-I data, 40 patients (65.5%) described their prolapse as ‘very much’ or ‘much’ better after a median follow-up time of five years (Table IV).

**Table IV:** Comparison of pre-operative and last PFDI-20 results with medium-long term patient satisfaction.

	Pre-operative	Last follow-up (years) 5 (1-12)	P-value
POPDI-6 score	33.3 (0-62.5)	0 (0-79.2)	0.001
CRADI-8 score	21.9 (0-62.5)	0 (0-25)	<0.001
UDI-6 score	50 (4.2-83.3)	8.3 (0-79.1)	<0.001
Total PFDI-20 score	119.7 (8.4-161.4)	30.2 (0-91.7)	<0.001
PGI-I scores	-		N/A
1		24 (39.3)	
2		16 (26.2)	
3		12 (19.7)	
4		4 (6.6)	
5		4 (6.6)	
6		0	
7		1 (1.6)	

Note: POPDI: pelvic organ prolapse distress inventory; CRADI: colorectal anal distress inventory; UDI: urinary tract distress inventory; PFDI: pelvic floor distress inventory. PGI-I, patient global impression of improvement. Continuous data are presented as median (min-max) values and categorical data are presented as frequency (percentage).

The total and all subdomain scores of the PFDI- 20 recorded at the last visit were significantly improved when compared to the pre-operative evaluation (P<0.001) (Table IV).

In a further analysis, follow-up outcomes of patients who underwent concomitant vaginal wall repair (n=37) and who did not (n=24), were also compared (Table V). Accordingly, the median PFDI-20 and PGI-I scores at the last visit were similar between the groups (33.3 vs. 34.4, P=0.460 and 1.5 vs. 2, P=0.182; respectively). There was one patient in each group who had isolated anterior prolapse recurrence.

**Table V:** Comparison of PFDI-20 results at last follow-up and medium-long term patient satisfaction between patients who underwent laparoscopic smSHP with or without vaginal wall repair.

	Concomitant vaginal wall repair (n=37)	No vaginal wall repair (n=24)	P-value
POPDI-6 score	0 (0-79.2)	12.5 (0-37.5)	0.538
CRADI-8 score	0 (0-25)	3.1 (0-9.4)	0.494
UDI-6 score	25 (0-79.1)	4.2 (0-20.8)	0.974
Total PFDI-20 score	33.3 (0-91.7)	34.4 (0-38.5)	0.460
PGI-I	1.5 (1-7)	2 (2-3)	0.182

Note: POPDI: pelvic organ prolapse distress inventory; CRADI: colorectal anal distress inventory; UDI: urinary tract distress inventory; PFDI: pelvic floor distress inventory. PGI-I, patient global impression of improvement. Data are presented as median (min-max) values.

## Discussion

The present study was conducted to assess medium to long term patient reported outcomes and recurrence rates of laparoscopic smSHP procedure. According to the results, it was found that the laparoscopic smSHP procedure provided long- standing patient satisfaction at medium to long- term follow-up. The symptomatic recurrence rate was relatively low (13.1%), and the re-operation rate was 3.2%. There was no mesh related adverse events.

A recent meta-analysis compared uterine preservation surgeries and hysterectomy techniques for the treatment of uterine prolapse and reported that uterine preservation surgeries improved operating time and blood loss without a significant change in short-term prolapse outcomes (Meriwether et al., 2018). In the comparison of mesh SHP and vaginal hysterectomy with uterosacral suspension, the level of point C and total vaginal length favoured uterine preservation surgery. In addition, the authors made a sub-group analysis and compared mesh SHP with hysterectomy plus mesh SHP; estimated blood loss, operating time, surgical cost, and mesh exposure all favoured uterine preservation, without an increased risk of recurrence (Meriwether et al., 2018). Recently, we reported the comparison of long-term results of laparoscopic SHP and vaginal hysterectomy and McCall culdoplasty and suggested that laparoscopic SHP was associated with significantly lower symptomatic recurrence and repeat surgery rates and better pelvic floor function in women younger than 60 years of age (Şükür et al., 2020).

In the present study, the PGI-I scores revealed that 65% of the patients felt very much or much better in the long-term, which is relatively lower than the literature (Rahmanou et al., 2014; Jefferis et al., 2017). However, the PGI-I scores in the present study were obtained at the last follow-up visits and reflect the long-term impression of patients. Besides satisfaction, most patients also reported long-standing improvement in their pelvic floor dysfunction. In the comparison of preoperative and medium-long term postoperative status of pelvic floor dysfunction the PFDI-20, not only the overall score, but all of the subdomain (POPDI-6, CRADI-8, UDI-6) scores were found to be significantly improved with our technique. Thus, laparoscopic smSHP might be an acceptable option as it seems successful and safe with a low risk of adverse events and low repeat surgery rates.

The long-term repeat surgery rate was found to be quite low (3.2%) with the laparoscopic smSHP technique. Previous studies of dual-mesh SHP procedures reported repeat surgery rates between 0% to 13.6% in both short- and long- term follow-up studies (Rahmanou et al., 2014; Jefferis et al., 2017; Lone et al., 2018; Izett-Kay et al., 2020). Jefferis et al. (2017) reported repeat apical prolapse surgery in 2.8% and repeat anterior repair in 7.1%, at 10-years follow-up. Recently, Daniels et al. (2020) reported 15.9% symptomatic recurrence, mostly occurring within two years following laparoscopic smSHP. However, almost all of those patients underwent concomitant vaginal repair, in which significant synthetic mesh usage was reported. The authors suggested an evident association with anterior synthetic mesh utilization and anterior prolapse recurrence. The damage in the yet created mesh site anteriorly probably might have necessitated higher rates of anterior prolapse recurrence. In our single mesh technique, native anterior and or posterior vaginal wall repairs were performed concomitantly before laparoscopic apical suspension in patients with concurrent stage 3-4 anterior and or posterior prolapse. We observed low symptomatic recurrence and repeat surgery rates at medium to long-term follow-up. Similarly, Jan et al. (2018) describing the same simplified single mesh technique, found no apical recurrence in 25 patients, but they reported three mild recurrences, managed with expectant management or minor surgeries.

The presented surgical technique has a theoretical advantage. As the mesh does not wrap the cervix, this technique may potentially allow cervical dilation in case of late miscarriage or vaginal delivery. However, this statement should be interpreted with caution. We do not support vaginal delivery after SHP, but in case of emergencies or contraindications for caesarean section, smSHP may enable vaginal delivery. Additionally, hysterectomy may be easier if required in the future.

The major complication rate of laparoscopic SHP has been reported to be low (Rahmanou et al., 2014; Jefferis et al., 2017; Jan et al., 2018; Daniels et al., 2020). We observed minor and major complications in 6.6% of cases and there was no mesh related adverse events. Supportingly, Daniels et al. (2020) also did not experience mesh erosion using a similar simplified technique. Moreover, the presented technique may decrease the risk of broad ligament bleeding or bladder injury during surgery and mesh removal due to pain (Jefferis et al., 2017; Izett-Kay et al., 2020). Nevertheless, the rate of concurrent vaginal wall repair was relatively higher with this technique when compared to dual- mesh surgeries (Kupelian et al., 2016; Jefferis et al., 2017). Although the patient reported outcomes were similar between women who underwent concomitant vaginal surgery and those who did not, it is noteworthy to mention that the sample size was relatively low to get a clear conclusion.

The major strength of our study is the presentation of medium-long-term follow-up results based on patient reported outcomes. There are some limitation which should be taken into consideration while interpreting the results. The retrospective design may bring selection and misinformation bias together. Another limitation is its relatively small sample size. Additionally, objective outcome determined by POP-Q examination was not available in all women. Finally, the absence of a control group should also be noted.

In conclusion, laparoscopic smSHP may be an acceptable option in the surgical management of apical prolapse with sufficient long-term patient satisfaction, low repeat surgery and adverse event rates. After appropriate counselling, laparoscopic smSHP may be offered to patients who prefer to preserve their uteri with the advantage of minimising mesh use. However, randomised controlled trials directly comparing short- and long-term results of smSHP and dual-mesh surgeries are urgently needed to identify the best surgical treatment.
